# Association of rs2073618 polymorphism and osteoprotegerin levels with hypertension and cardiovascular risks in patients with type 2 diabetes mellitus

**DOI:** 10.1038/s41598-023-44554-0

**Published:** 2023-10-14

**Authors:** A. Naga Sailaja, Nivedita Nanda, B. S. Suryanarayana, G. K. Pal

**Affiliations:** 1 Department of Biochemistry, Jawaharlal Institute of Postgraduate Medical Education and Research (JIPMER), Puducherry, 605 006 India; 2 Department of Medicine, Jawaharlal Institute of Postgraduate Medical Education and Research (JIPMER), Puducherry, India; 3 Department of Physiology, Jawaharlal Institute of Postgraduate Medical Education and Research (JIPMER), Puducherry, India; 4 Present Address: AIIMS, Patna, India

**Keywords:** Physiology, Cardiology, Endocrinology, Health care

## Abstract

There are reports of link of osteoprotegerin (OPG) gene polymorphism to type-2 diabetes (T2D) and hypertension (HTN). The objective of the study was to assess the allele frequency of OPG (rs2073618) gene polymorphism and its association with heart rate variability (HRV) and blood pressure variability profile as CVD risks in diabetes mellitus patients with hypertension undergoing treatment. T2D patients on treatment without hypertension (n = 172), with hypertension (n = 177) and 191 healthy volunteers were recruited for the study. Their blood pressure variability including baroreflex sensitivity (BRS), heart rate variability (HRV), OPG, insulin, lipid profile, receptor-activator for NFkB (RANK), receptor-activator for NFkB-Ligand (RANKL), and tumor necrosis factor-α (TNF-α) were estimated. Allele frequency of OPG (rs2073618) gene polymorphism was assessed from the DNA samples. BRS and HRV indices were decreased, and RANKL/OPG and TNF-α were increased in T2D and T2D + HTN groups, respectively compared to healthy control group. The reduction in BRS was contributed by increased inflammation and reduced SDNN of HRV in GG genotype in T2D + HTN. In GG + GC subgroup, it was additionally contributed by rise in RANKL/OPG level (β − 0.219; *p* 0.008). Presence of mutant GG genotype contributed to the risk of hypertension among T2D patients (OR 3.004) as well as in general population (OR 2.79). It was concluded that CV risks are more in T2D patients with HTN expressing OPG rs2073618 gene polymorphism.

## Introduction

According to World Health Report 2002, cardiovascular diseases (CVD) will be the largest cause of death and disability by 2020 in India. Nearly half of these deaths are likely to occur in young and middle-aged individuals (30–69 years) especially who have diabetes and or hypertension or are genetically susceptible to develop diabetes and or hypertension^1,2^. Diabetes mellitus and hypertension are two major established risk factors for CVD^3^. Further, coexistence of hypertension with insulin resistance increases the risk of target organ damage and clinical cardiovascular accidents^4^. Nevertheless, not all patients with diabetes mellitus develop hypertension at least in the first 5 years^5^. However, a quite significant number of patients with diabetes develop hypertension quite early after acquiring the disease^6^. Genetic susceptibility for hypertension has been proposed to play important role in nearly 50% of insulin resistant individuals to develop hypertension, in the early phase of the disease even after receiving standard antidiabetic treatment^7^. Till date, no study has been conducted to assess the pathophysiological difference in the CVD risk profile of diabetes patients with or without having hypertension.

Vascular calcification, a factor common to both CVD and hypertension is no longer considered as age related phenomenon^8^. Irrespective of the site and degree of involvement, the vascular calcification is a strong independent predictor of cardiovascular mortality^9^. The intimal calcification is associated with atherosclerosis while medial calcification seen in ageing is associated with arterial stiffening leading to reduced vascular compliance^8^. Arterial stiffening due to atherosclerosis accelerated by calcification is a known pathophysiological mechanism of hypertension^10^. Osteoprotegerin (OPG) is a biomarker of vascular calcification and associated with CVD. OPG is a decoy receptor for receptor-activator for NFkB-ligand (RANKL), which has been implicated in pathophysiology of CVD. OPG has also been linked to myocardial stiffness^11^, hypertension and diabetes^12^. Recently we have reported the link of OPG to CVD risk in diabetes^13^.

The *TNFRSF11B* gene at 8q24.12 of chromosome 8 encodes OPG in humans. There are several reports of single nucleotide polymorphisms (SNP) for OPG gene, linked to DM and CVD^14^. Among these, the 1181 G > C SNP polymorphism has been particularly associated with CVD, left ventricular hypertrophy in essential hypertension^15^ and abnormal coronary arteries^16^. The rs2073618 of OPG is an exon variant with G > C transversion at exon 1. This leads to change in the third amino acid of the signal peptide from lysine (AAG), into asparagine (AAC). Polymorphism at exon region can influence splicing by affecting the binding sites of enhancers and silencers thus affecting protein level in circulation. However, studies relating RANK/RANKL/OPG level or associated gene polymorphisms in this pathway, in relation to blood pressure alteration, are scarce in Indian population. We hypothesize that any alteration in the levels of receptors such as RANK and OPG; their major ligands such as RANKL and TNF-alpha; or the corresponding allele polymorphism of OPG could play a pivotal role in insulin resistance associated hypertension and subsequent increase in CV risks in the Southern Indian (Tamil) population.

Sympathetic nervous system (SNS) activation and retrograde inflammation are two major mechanisms in the genesis of hypertension^17–19^, insulin resistance^20,21^ as well as CVD^22,23^. Blood pressure variability (BPV) and heart rate variability (HRV) are two sensitive methods of measurements of CV risks in health and disease^24–26^. Decreased baroreflex sensitivity (BRS) an important parameter of BPV and decreased HRV as the marker of reduced cardiovagal modulation are the recently established indices of CVD risks in diabetes and hypertension. As BRS is influenced by mechanical properties of vascular wall, high vascular transmural pressure could alter the BRS and influences the autonomic regulation of blood pressure in diabetes with hypertension. Recently we have reported the plausible role of OPG in decreased cardiovagal modulation in diabetes^13^. Also, we have reported the link OPG with decreased BRS in patients with diabetes receiving oral anti-diabetic drugs^27^. However, the CVD risks has not been assessed in patients with diabetes with or without hypertension expressing SNP of OPG gene. Therefore, the primary objective of the study was to assess the allele frequency of OPG (rs2073618) gene polymorphism in diabetes mellitus patients under treatment with hypertension compared with similar age, gender and ethnicity matched diabetes patients without hypertension. Also, we have assessed the HRV and BPV profile as CVD risks and their association with gene polymorphism of OPG in these patients.

## Materials and methods

### Study design

This was a single center cross sectional comparative study in the Southern Indian (Tamil) population consisting of three groups. The study was first approved by an institution research review board and the Ethics Committee (Human studies: JIP/IEC/2018/305) prior to commencement. Verbal approval followed by written informed consent was obtained from the participants before recruitment.

#### Participants

Participants were divided into three groups: control group, test group 1 and test group 2, based on the following inclusion criteria.

#### Control group

The healthy volunteers with no history of diabetes mellitus or hypertension were recruited as subjects in control group.

#### Test groups

For test groups, individuals in the age 20–60 years, diagnosed with diabetes mellitus and under treated with metformin and glimepiride hypoglycemic agents combination therapy for at least for two years, were recruited consecutively from outpatient clinic of medicine dept. Further these patients with T2D were segregated as test group 1 and test group 2 based on absence and presence of hypertension respectively. Thus, ***Test group 1*** consisted of Tamil patients diagnosed as diabetes mellitus without hypertension, and ***Test group 2*** consisted of Tamilian Diabetes mellitus patients diagnosed with hypertension as per JNC guidelines or New ACC/AHA High blood pressure guidelines^28^, (SBP > 140 Hg or DBP > 90 Hg) and under treatment with amlodipine and enalapril.

### Study procedures

**Figure 1:**
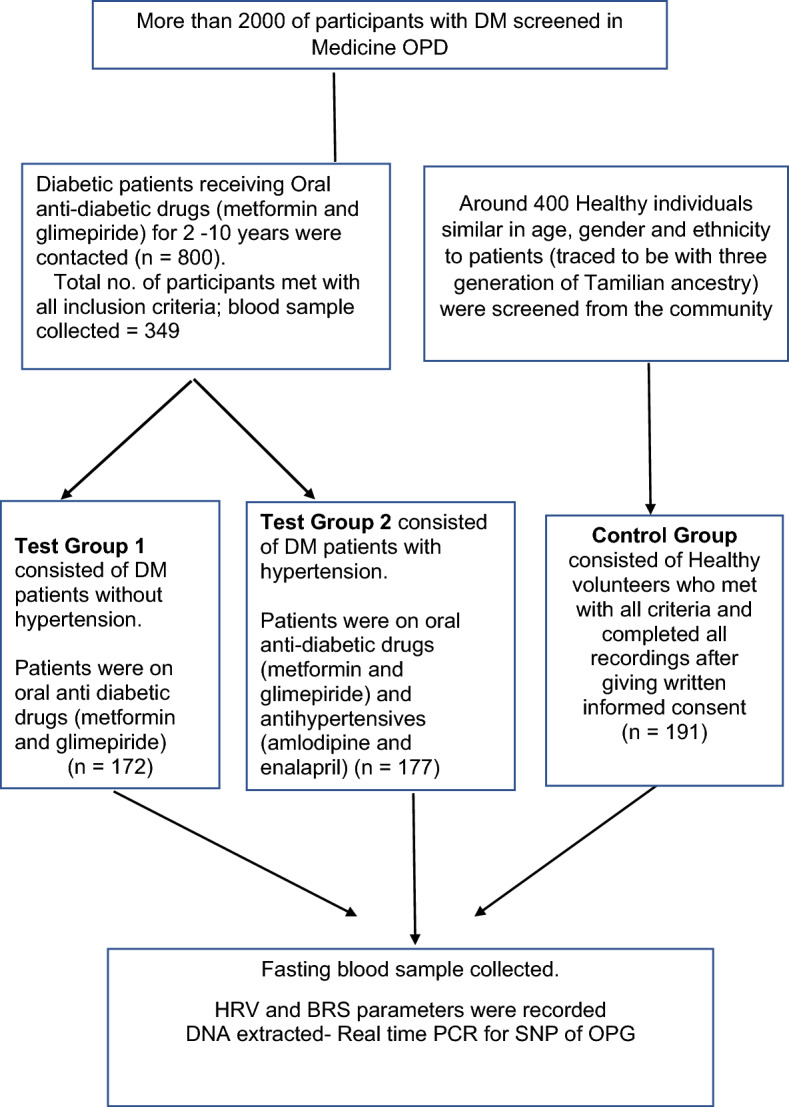
Flow chart of participants’ recruitment.

The participants were briefed about the study protocol and their written informed consents were obtained (Fig. 1). Basal demographic and anthropometric data were collected, and the personal history was noted using a structured data sheet. The next day morning their fasting blood samples were collected following which the blood pressure, HRV and BRS recordings were taken as per the standard procedures described earlier^27^.

For the recording of short-term HRV, ECG electrodes were used. The RR tachograms were reconstructed using BIOPAC MP 100 data acquisition system (BIOPAC Inc., USA). The frequency-domain indices of HRV included total power (TP), low frequency power expressed in normalized units (LFnu), high frequency power expressed in normalized units (HFnu), and ratio of low-frequency to high-frequency power (LF-HF ratio); and the time-domain indices included mean standard deviation of RR intervals (SDNN), square root of the mean of the sum of the squares of the differences between adjacent RR interval (RMSSD), adjacent RR interval differing more than 50 ms (NN50), and NN50 counts divided by all RR intervals (pNN50).

For BRS measurement continuous blood pressure variability method by Finapres (Finometer version 1.22a, Finapres Medical Systems, Amsterdam, The Netherlands) was used which analyses the BPV in a non-invasive manner using the principle of finger plethysmography.

### Assessment of biochemical parameters

Part of the fasting blood sample was collected in EDTA tube for DNA extraction and rest was collected in serum separator tube. Same day serum was processed for fasting glucose and lipid profile assay and rest was stored in aliquots for ELISA at − 40 °C. Fasting serum glucose (FSG), direct LDL cholesterol and lipid profile were measured by an automated AU5800 chemistry analyzer using commercial kits (Beckman Coulter AU5800, Beckman Coulter Inc, Brea, California, USA).

### Genotyping assay

Whole blood samples collected in EDTA tube were used for extraction of genomic DNA by standard phenol–chloroform method. DNA was quantified using a nanodrop. Genotyping was done using predesigned TaqMan probes and primers for OPG rs2073618 (Thermofischer scientific assay ID C_1971047_40), using real time PCR as per manufacturer’s instruction.

### Metabolic, inflammatory, and cardiovascular risk markers parameters

TNF-α (Diaclone, France), Insulin (Calbiotech, USA), RANK (Elabscience, USA), RANKL (Elabscience, USA) and OPG (Fine test, China) were estimated by an enzyme-linked immunosorbent assay based on the principle of sandwich ELISA and the mean value was determined from the standard dose–response curves. HOMA-IR was calculated using formula HOMA-IR = Glucose X Insulin/405^29^. The inter-assay coefficients of variation for ELISA parameters for RANKL, RANK, TNF-α, OPG and insulin were < 6%, < 7%, < 11%, < 5.5% and < 6.1% respectively. The intra-assay of coefficients of variation for ELISA parameters for RANKL, RANK, TNF-α, OPG and insulin were < 6%, < 5.5%, < 3.2%, < 5.5% and < 3.5% respectively.

### Statistics

All statistical analyses in the present study were performed using the Statistical Package for Social Sciences Software Version 20.0 (IBM PASW Statistics), Graph Pad Prism version 8.00, Graph Pad Instat Version 3.10 (San Diego, USA) and SNPstat online software. The Shapiro Wilk test tested the normality of the data, and accordingly, appropriate parametric or non-parametric tests were used. The non-Gaussian data is expressed as median with Inter-Quartile Range (IQR). Kruskal–Wallis test ANOVA followed by Dunn’s Bonferroni post hoc test was used to compare the quantitative data among three groups. Spearman correlation was used to test for association among quantitative variables followed by multiple regression analysis was used to assess the independent contribution of variables. Hardy–Weinberg equilibrium (χ^2^) was tested on genotype frequencies by chi-square test. Direct gene counting was utilized for the calculation of allele frequencies. Differences in genotype distributions and allele frequencies in the groups were compared using the chi-square test. The association of genotypes with T2D with hypertension was analyzed under four genetic models (dominant, recessive, codominant, and homozygotic) using the logistic regression. The results were expressed as a percentage. The association between the disease and genotype was checked by chi-square test and expressed as odds ratio (ORs) with 95% Confidence Intervals (CIs). For all the statistical tests, a *p*-value < 0.05 was considered significant.

### Ethics approval

This study was approved by the JIPMER Institute Ethics Committee [JIP/IEC/2018/305]. All methods in the study were performed in accordance with the Helsinki declarations. All data of the study were anonymised and coded before its use.

### Consent to participate

Written informed consent to use the biological sample and case history was obtained from each participant after explaining the purpose, and procedure of the study in their vernacular language.

## Results

### Anthropometric and demographic findings

The distributions of age, BMI, blood pressure variability parameters, heart rate variability parameter, metabolic-calcification and inflammation markers are shown in Table 1. There were no significant differences found between the groups in their baseline characteristics such as age and BMI.

**Table 1:** Comparison of demographic, anthropometric indices, blood pressure and blood pressure variability (BPV) parameters, heart rate variability (HRV) parameters among healthy control, diabetes mellitus (DM) patients on treatment without hypertension and with hypertension (HTN).

Variables	Control group	Test group (1)	Test group (2)	*P* values
	(Healthy control) (n = 191)	(DM without HTN) (n = 172)	(DM with HTN) (n = 177)	
Age (Years)	51 (43–59)	50 (46–56)	52 (47–57)	0.349
BMI (Kg/m^2^)	25.65(23.93–28.24)	25.80(23.17–28.67)	26.63(24.33–28.88)	0.133
BPV parameters				
BHR (beats per min)	75 (70–83)	77 (69.25–84)	80 (72–88.50)**, ^#^	0.001
SBP (mmHg)	122 (112–128)	121 (112–129)	133 (122.50–143)***,^###^	< 0.001
DBP (mmHg)	79 (72—87)	78 (72–84)	83 (78–88 )***,^##^	< 0.001
RPP (mmHg/min)	91.63 (80.30–104.58)	91.67 (80.45–104.49)	105.57 (93.70–118.30)***,^###^	< 0.001
BRS (ms/mmHg)	16.06 (13.55–19.356)	13.02 (8.79–16.67)	6.27 (4.31–8.27)***,^###^	< 0.001
HRV parameters				
TP(ms^2^)	1328 (839—2042 )	885(615.50–1185.75)***	469 (368–655 )***,^###^	< 0.001
LFnu	46.20 (35.92–58.95)	52.32(43.31–59.91)*	64.00 (52.00–72.50)***,^###^	< 0.001
HFnu	53.799 (41.35–64.07)	47.67(40.08–56.68)*	6.00 (27.50–48.00)***,^###^	< 0.001
LF/HF	0.85 (0.56–1.41)	1.09(0.76–1.49)*	1.79 (1.09–2.70) ***,^###^	< 0.001
SDNN (ms)	43.70 (32.70–57.00)	31.30 (23.77–42.00)***	22.70 (15.13–28.00)***,^###^	< 0.001
RMSSD(ms)	42.00 (20.00–90.00)	26.00 (18.85–34.30)***	15.80 (10.00–24.05)***,^###^	< 0.001
NN50	20.40 (13.45–25.90)	12.00 (9.00–19.00)***	8.00 (5.00–11.00)***,^###^	< 0.001
PNN50	25.00 (17.00–37.70)	8.00 (5.00–9.97)***	2.20 (2.00–4.13)***,^###^	< 0.001

Data is expressed as Median with interquartile range. Comparison among the groups was done by Kruskalwallis test. *P Value < 0.05; ** *P* Value < 0.01; *** *P* Value < 0.001 was considered significant, when comparison is done with control; #*P* Value < 0.05; ^##^ *P* Value < 0.01; ^###^ *P* Value < 0.001 was considered significant, when comparison is done with patients with DM without hypertension. BMI: Body mass index; BHR: Basal heart rate; SBP: Systolic blood pressure; DBP: Diastolic blood pressure; RPP: Rate pressure product; BRS: Baroreflex sensitivity; TP: Total power of HRV, LFnu: Normalized low-frequency power of HRV; HFnu: Normalized high-frequency power of HRV; LF/HF: Ratio of LFnu and HFnu; SDNN: Standard deviation of normal to normal interval; RMSSD: Square root of the mean of the sum of the squares of the differences between adjacent NN intervals. NN50: the number of interval differences of successive NN intervals greater than 50 ms; PNN50; the proportion derived by dividing NN50 by the total number of NN intervals.

### BPV, HRV and circulating biomarkers findings

A significant rise was observed in the BHR, SBP and DBP while BRS was reduced in T2D with hypertension patients compared to T2D patients and healthy controls (Table 1). Among the HRV indices TP, SDNN, RMSSD, NN50 and pNN50 were significantly less in T2D patients with hypertension (Table 1) compared to T2D patients and healthy control. LFnu was significantly higher, HFnu was considerably lower, and LF/HF ratio was significantly higher in T2D patients with hypertension (Table 1).

### Metabolic and inflammatory markers and atherogenic indices

The serum concentration of glycemic parameters, lipid profile and atherogenic indices among study participants are presented in Table 2. There was no difference in FSG and HbA1c levels between the two patient groups though it was more compared to healthy control. Insulin and HOMA-IR was while TC and HDL cholesterol were low in T2D + HTN group compared to T2D and healthy control group. LDL cholesterol was similar across all groups while triglyceride was high only in T2D + HTN group.

**Table 2:** Comparison of metabolic, inflammatory and calcification markers among healthy control, diabetes mellitus (DM) patients on treatment without hypertension and with hypertension (HTN).

Variables	Control group	Test group (1)	Test group (2)	*P* values
	(Healthy control) (n = 191)	(DM without HTN) (n = 172)	(DM with HTN) (n = 177)	
Metabolic parameters for Glycemic profile				
FSG (mg/dL)	84 (76–97)	133 (106.25–165) ***	133 (117.50–187.50)***	< 0.001
HbA1c (g%)	5.4 (5.20–5.60)	7 (6.44–7.15) ***	7 (6.41–7.31)***	< 0.001
Insulin (μU/mL)	9.71 (6.85–13.16)	22.56 (9.55–38.64) ***	26.11 (17.73–49.03)***,^###^	< 0.001
HOMA-IR	1.97 (1.36–2.84)	5.85 (2.71–12.19) ***	8.29 (5.10–17.06) ***,^###^	< 0.001
Metabolic parameters for lipid profile				
TC(mg /dL)	175 (157 -194)	157 (135.25–183)***	147 (126.50–177) ***	< 0.001
HDL-C(mg /dL)	44 (40–49)	37 (33.25–43.75) ***	37 (32 -40.50) ***	< 0.001
LDL-C(mg /dL) 103.40(88.80–117.20)	103.40 (88.80-117.20)	94.50 (73.00–117.00)**	96.00 (79.50–122.00)	0.005
TG(mg /dL)	128 (103 -150)	135.50 (99.25–180)	145 (110.50–189.00)**	0.004
VLDL-C(mg /dL)	25.60 (20.60–30.0)	27.10(19.85–36.0)	29 (22.10–37.80) **	0.004
Inflammatory & calcification markers				
TNF-α (pg/mL)	4.81 (2.72–7.19)	14.58 (9.48–20.83)***	20.24 (13.00–27.29)***,^###^	< 0.001
OPG (pg/mL)	181.76 (134 -300.25)	215.79 (131.99–306.99)	260.51 (199.38–349.92)***,^###^	< 0.001
RANK (ng/mL)	0.61 (0.50–0.77)	1.15 (0.61–2.43)***	0.97 (0.59–1.77)***	< 0.001
RANKL (pg/mL)	7.13(5.69–9.76)	10.14(6.40–19.77)***	11.91(6.10–24.65) ***	< 0.001
RANKL/OPG	0.03 (0.01–0.04)	0.06 (0.02–0.12) ***	0.05 (0.02–0.08) ***	< 0.001

Data is expressed as Median with interquartile range. Comparison among the groups was done by Kruskalwallis test. *P Value < 0.05; ** *P* Value < 0.01; *** *P* Value < 0.001 was considered significant, when comparison is done with control; ^#^*P* Value < 0.05; ^##^ *P* Value < 0.01; ^###^ *P* Value < 0.001 was considered significant, when comparison is done with patients with DM without hypertension. FSG: Fasting serum glucose; HbA1c: Glycated haemoglobin; HOMA-IR: homeostatic model assessment of insulin resistance; TC: Total cholesterol; TG: Triglyceride; HDL: High density lipoprotein; LDL: Low density lipoprotein; VLDL: Very low-density lipoprotein; Non HDL-C: Non HDL cholesterol; AIP: Atherogenic index of plasma = log_10_[TG/ HDL- C]; TNF- α: tumor necrosis factor alpha; OPG: Osteoprotegerin; RANK: Receptor activator of NfkB; RANKL: Receptor activator of NfkB ligand.

The median TNF α, OPG and RANKL/OPG levels were higher in T2D with hypertension group (Table 2) compared to T2D as well as control groups. There was no difference in RANK and RANKL levels when compared between two test groups. The correlated of these markers were checked with BRS for both test groups (Table 3).

**Table 3:** Spearman correlation of BRS with various parameters in treated T2D patients with and without hypertension.

Parameters	Test Group (1)		Test Group (2)	
	DM without HTN (n = 172)		DM with HTN (n = 177)	
	*r*	*P*	*r*	*P*
BHR	− 0.168	0.028	− 0.312	0.000
SBP	− 0.010	0.901	− 0.173	0.022
RPP	− 0.127	0.098	− 0.342	0.000
HbA1c	− 0.065	0.532	− 0.233	0.019
HOMA-IR	0.032	0.700	0.019	0.807
LF/HF	− 0.080	0.294	− 0.288	0.000
TP	0.050	0.514	0.309	0.000
SDNN	0.083	0.281	0.440	0.000
RMSSD	0.064	0.407	0.333	0.000
OPG	− 0.129	0.093	− 0.366	0.000
TNF α	− 0.042	0.587	− 0.497	0.000
RANK	0.211	0.006	0.181	0.017
RANKL	0.049	0.527	− 0.360	0.000
RANKL/OPG	0.143	0.063	− 0.193	0.011

RPP: rate pressure product; BHR: basal heart rate; SBP: systolic blood pressure, FBG: fasting blood glucose; TG: triglyceride; HDL: high density lipoprotein; AIP: atherogenic index of plasma; SDNN: standard deviation of normal-to-normal interval; RMSSD: square root of the mean squared differences of successive normal to normal intervals; CO: cardiac output; OPG: Osteoprotegerin, *P Value < 0.05 was considered significant.

### Genotyping and genetic model analysis

The genotype and allelic frequency distribution of OPG rs2073618 gene polymorphism is shown in Table 4 between T2D patients with hypertension and healthy control and in Table 5 between T2D patients with and without hypertension. For calculation of risks, we used logistic regression analysis using dominant model. The odds for risk of T2D + HTN in general population was 2.79 (1.44–5.21) as the comparison was between T2D + HTN and healthy control. The odds for risk of HTN among diabetic patients was 3.004 (1.55–5.58) as the comparison was between T2D + HTN and T2D without hypertension. Among the T2D + HTN patients, the recessive model was significant when compared with healthy control, and both the recessive and dominant models were significant when compared to T2D patients (Tables 4 and 5 respectively). Therefore, among the T2D and T2D + HTN patients, correlation of decrease in BRS was assessed for various parameters (Table 3). Subsequently contribution of different risk factors towards decreased BRS was analyzed for GG and GG + GC genotype subgroups separately for the T2D + HTN patients (Table 6). In GG genotype subgroup, BRS was contributed by rise in inflammation and reduction in SDNN. In GG + GC genotype subgroup, apart from TNF α and SDNN, the rise in RANKL/OPG level was another independent contributor for BRS (Table 6).

**Table 4:** Genotype and allelic frequency distribution of OPG (TNFRSF11B) rs2073618 gene polymorphism between T2D patients with HTN (n = 177) and healthy control (n = 191).

Genotypes& alleles	T2D with HTN (n = 177)	Healthy control (n = 191)	OR (95% CI)	*P* value
Genotypes				
CC (2)	52 (29.4%)	71 (37.2%)	Ref	––
GC (1)	81 (45.8%)	98 (51.3%)	1.12 (0.70–1.77)	0.60
GG (0)	44 (24.9%)	22 (11.5%)	2.79 (1.44–5.21)	0.0014
Alleles				
C	185 (52%)	240 (62%)	Ref	0.003
G	169 (48%)	142 (38%)	1.54 (1.15–2.07)	
Genetic models				
Dominant				
CC	52 (29.4%)	71 (37.2%)	Ref	0.11
GC + GG	125 (70.6%)	120 (62.8%)	1.42 (0.92–2.20)	
Recessive				
CC + GC	135 (79.9%)	168 (88%)	Ref	0.0008
GG	34 (20.1%)	23 (12%)	2.54 (1.45–4.45)	
Homozygotic				
CC	52 (29.4%)	71 (37.2%)	Ref	0.0014
GG	44 (24.9%)	23 (11%)	2.79 (1.44–5.21)	

Genetic model reference; OR: Odds ratio; Ref: Reference; CI: Confidence interval.

**Table 5:** Genotype and allelic frequency distribution of OPG (TNFRSF11B) rs2073618 gene polymorphism between T2D patients with HTN and without HTN.

Genotypes & alleles	T2D with HTN (n = 177)	T2D without HTN(n = 172)	OR (95% CI)	*P* value
Genotypes				
CC (2)	52 (29.4%)	71(41.3%)	Ref	–
GC (1)	81 (45.8%)	81 (47.1%)	1.36 (0.86–2.18)	0.13
GG (0)	44 (24.9%)	20 (11.6%)	3.004 (1.55–5.58)	0.0006
Alleles				
C	185 (52%)	223 (64%)	Ref	0.0008
G	169 (48%)	121 (36%)	1.68 (1.24–2.28)	
Genetic models				
Dominant				
CC	52 (29.4%)	71 (41.3%)	Ref	0.02
GC + GG	125 (70.6%)	101 (58.7%)	1.69 (1.08–2.63)	
Recessive				
CC + GC	133 (75.1%)	152 (88.4%)	Ref	
GG	44 (24.9%)	20 (11.6%)	2.51 (1.14–4.48)	0.0001
Homozygotic				
CC	52 (29.4%)	71 (41.3%)	Ref	
GG	44 (24.9%)	20 (11.6%)	3.004 (1.55–5.58)	0.0006

Genetic model reference; OR: Odds ratio; Ref: Reference; CI: Confidence interval.

**Table 6:** Multiple regression analysis to assess the independent association of BRS (as dependant variable) with metabolic, inflammation and calcification markers (as independent variables) in patients with hypertension in recessive model (GG population) and dominant model (GC + GG population).

Independent Variable	GG (n = 44)				GC + GG (= 125)			
	Standardized regression coefficient β	95% CI		*P* value	Standardized regression coefficient β	95% CI		*P* value
		UL	LL			UL	LL	
BMI	0.085	− 0.144	0.279	0.520	0.094	− 0.301	0.079	0.250
RANKL/OPG	− 0.179	− 23.216	4.646	0.185	− 0.219	− 22.327	− 3.353	0.008
TNF α	− 0.414	− 0.177	− 0.037	0.004	− 0.367	− 0.221	− 0.084	0.000
SDNN	0.356	0.021	0.151	0.011	0.218	0.014	0.095	0.009

BRS: Baroreflex sensitivity; BMI: Body mass index; RANKL: Receptor activator of NfkB ligand OPG: Osteoprotegerin; TNF α: tumor necrosis factor alpha; SDNN: Standard deviation of normal-to-normal interval. The *p* value < 0.05 was considered significant.

## Discussion

The purpose of a genetic study is mainly to identify genetic risk factors for common and complex diseases^30^. Cardiovascular disease is one such complex disease and considered a polygenetic disease. The prevalence of CVD and its risk factors such as diabetes and hypertension are high in Indian population. There are previous reports of osteoprotegerin gene polymorphism rs2073618^31^ linked to cardiovascular disease^14^ in the non-Indian population. However, the reports on single nucleotide gene polymorphism for RANKL-RANK-Osteoprotegerin pathway from Indian subcontinent is lacking. In our previous study, we reported the association of higher serum osteoprotegerin level among patients with diabetes mellitus^13^. We also reported the association of T950C gene polymorphism for osteoprotegerin among females with gestational diabetes mellitus^32^. Diabetes mellitus being a major risk factor for CVD, we hypothesize that those with osteoprotegerin gene polymorphism might be at greater risk to develop diabetes and hence CVD. However, among diabetes around 50% of patients develop hypertension during their disease despite treatment, which further elevates their CV risk^5^. This leads to the original question as to whether there is a genetic association for developing hypertension quite early among patients with insulin resistance and thereby adding to higher CV risk while the others are protected. To the best of our knowledge, there are no reports on the link of polymorphisms in the OPG-RANK-RANKL genes among the diabetic population with and without hypertension. Therefore, the main objective of the present study was to assess the allele frequency of OPG (rs2073618) gene polymorphism in T2D patients under treatment with hypertension compared with T2D patients without hypertension.

In the present study we followed candidate gene approach for genetic association of OPG rs2073618 gene polymorphism (1181G > C) in the exon 1 region, with the cardiovascular risk pattern among patients with T2D and the risk of developing hypertension. We found a positive association of OPG rs2073618 gene polymorphism (1181G > C) in diabetes patients with hypertension as there was a significant over-representation of polymorphic GG genotype in patients when compared with healthy control (OR: 2.79; *p* = 0.0014; Table 4), and patients with diabetes mellitus without hypertension (T2D + HTN) (OR: 3.004; *p* = 0.0006; 5). These findings suggest that the 1181G > C variant causing rs2073618 gene polymorphism, could be associated with hypertension. In the model analyses, the recessive model (GG) was significant (*p* = 0.0008) (Table 4) compared with control while dominant model (GG + GC) was significant when compared to T2D alone (Table 5). This suggests that the risk of developing hypertension is conferred in a recessive model in the general population. However, the risk of hypertension increases several folds among diabetes patients if they carry OPG gene 1181G > C polymorphism even with single G allele. Therefore, G mutant allele for OPG rs2073618 gene polymorphism appears to be associated with risk factors which confer susceptibility to develop hypertension.

Both formation of atherosclerotic plaque and its rupture are associated with adverse cardiovascular events. Among several factors, vascular calcification especially the calcification at intima and media can significantly raise the risk for atherosclerotic plaque formation. When this is associated with a reduced vascular compliance leading to a rise in blood pressure it adds to the risk of plaque rupture and myocardial workload. Therefore, risk factors for vascular calcification significantly increase the CV risks. Baroreflex being the link among heart rate, BP and vascular tone, a reduced BRS is a sensitive noninvasive CV risk marker^25^. In the present study the basal heart rate, systolic and diastolic blood pressure, and rate pressure product (RPP) were significantly high in test group 2 (T2D with hypertension) compared to control and test group 1 (T2D without hypertension) (Table 1). There was a significant reduction in BRS (Table 1) in patients with T2D + HTN when compared to healthy control (*p* < 0.001) and compared to patients with T2D (*p* < 0.001). Reduced BRS is an indicator of central autonomic dysregulation, which in turn is also a marker of loss of elastic properties of arteries in general circulation^26,33,34^. In other words, the study group 2 patients with T2D + HTN are more prone to CV risks compared to control group as well study group 1 patients with T2D.

The increase in basal heart rate is an index of reduced vagal tone and tachycardia at rest is associated with CV morbidity and mortality^35^. Significant reduction in TP and time-domain indices of HRV parameters such as, SDNN and RMSSD indicate a significant reduction in the cardiac vagal modulation in both test group 1 and 2. The reduction in SDNN is a gold standard for medical stratification of cardiovascular risks^36^. In the present study, BRS had an independent association with SDNN, TNF α RANKL/OPG, in patients with T2D + HTN expressing mutant allele of OPG rs2073618 gene polymorphism in GG genotype subgroup and GC + GG genotype subgroup (Table 6). To the best of our knowledge, this is the first report on link of CV risk to OPG (TNFRSF11B) rs2073618 gene polymorphism in Indian population.

The findings of present study demonstrate that patients with diabetes associated hypertension have persistent signs of inflammation and vascular calcification and altered metabolic markers in the form of an atherogenic lipid profile (Table 2). RANKL/OPG significantly contributed to reduced BRS in the population expressing G allele (GG and GC genotype) but not with GG genotype alone suggesting that vascular calcification is not a major link for loss of autonomic control in GG genotype although they are at high risk of hypertension. Nonetheless, it also indicates that the population with GG and GC genotype are at risk of hypertension when one or multiple mechanisms are active such as vascular calcification, inflammation and altered heart rate variability. Therefore, this group (GG + GC) of individuals are at higher cardiometabolic risks.

In the present study, levels of OPG, RANKL and RANKL/OPG were all high in test groups 1 and 2 compared to healthy control. A high RANKL/OPG ratio is associated with arterial calcification as RANKL directly promotes smooth muscle cell calcification^37^. OPG plays a pivotal role in the RANKL-RANK-Osteoprotegerin pathway. Within physiological range it is protective but at a higher level, it is indicative of high rate of ongoing atherosclerosis. RANKL on the other hand is the ligand in this pathway and a rise in RANKL or the relative ratio of RANKL to OPG is suggestive of ensuing process of atherosclerosis and calcification. It is to be noted that in our study, all patients were under standard antidiabetic and anti-hypertensive treatment for a period of 2–10 years. The metformin treatment has a positive influence on OPG level while both metformin and amplodipin reduce RANKL level^38^. These effects are associated with reduced CV risk in treated patients with diabetes and/or hypertension. However, despite these beneficial effects, in our study, the median level of RANKL/OPG ratio was found to be higher in test group 2 suggesting a persistence of ongoing atherosclerotic process which was still higher compared to healthy controls (Table 2).

OPG, a member of the tumour necrosis factor α (TNF- α) receptor superfamily. It is secreted from various tissues including endothelium and smooth muscle cells. Its expression is regulated by various cytokines^39–41^. In the present study, TNF-α was significantly associated with G allele (both GG and GC genotype) and contributed to reduced BRS, which suggests the importance of retrograde inflammation in the onset of hypertension. Reports indicate that the inflammatory mediators like TNF-α can stimulate release of OPG from endothelial cells^42^. Also, high level of OPG can promote endothelial cell dysfunction and reduce the release of nitric oxide that could possibly be a mechanism of rise in blood pressure^43^. Thus, a high OPG level with persistent inflammation and reduced vagal tone for prolonged period can tilt the OPG/RANK/RANKL axis and may set the stage for development of hypertension.

There are previous reports indicating the association of rs2073618 polymorphism with T2D^10^ and morbidities associated with diabetes^44,45^. In our study, we demonstrated the association of rs2073618 polymorphism with diabetes associated hypertension. To the best of our knowledge, this is the first study to establish an association between SNP rs2073618 of OPG gene in South Indian Tamilians with T2D and hypertension. The mutant G allele occurred more frequently in patients with T2D + HTN. Also, both the GG and GC genotypes were both carrier of high risk for hypertension with an association with several other cardiometabolic and autonomic risk factors (Table 6). Thus, it is likely that hypertension is associated with inflammation, vascular calcification and reduced cardiovagal modulation in population expressing OPG rs2073618 gene polymorphism (1181 G > C) and the G allele is associated with increased risk of hypertension.

## Limitations of the study

The major limitation of the study is that the only one SNP for OPG gene was assessed in the present study, which was mainly due to the time and fund constrains of this PhD thesis work. Though the sample size for analysis of OPG gene polymorphism was adequate in the present study, the correlation of CV risk assessment would have still yielded a better level of significance with a higher sample size.

## Future scope

Our study provides a new insight into the risk of developing hypertension in diabetes associated with OPG gene polymorphism and the difference in the risk profile in terms of altered level of RANKL, osteoprotegerin, inflammation and autonomic control. This provides a direction towards future studies to be conducted in large cohorts and for the effective strategy of management of hypertension in diabetic subjects in the population with OPG 1181 G > C polymorphism as primary target as part of personalized medicine.

## Data Availability

The datasets analysed in this study are available with the corresponding author, which can be obtained on reasonable request.
